# Case Report: Successful Mechanical Thrombectomy in a Newborn With Basilar Artery Occlusion

**DOI:** 10.3389/fneur.2021.790486

**Published:** 2022-02-22

**Authors:** Christian Paul Stracke, Lukas Meyer, Wolfram Schwindt, Alexander Ranft, Ronald Straeter

**Affiliations:** ^1^Section of Interventional Neuroradiology, University Hospital Muenster, Muenster, Germany; ^2^Department of Diagnostic and Interventional Neuroradiology, University Medical Center Hamburg-Eppendorf, Hamburg, Germany; ^3^Department of Interventional Radiology and Neuroradiology, Klinikum Hochsauerland, Arnsberg, Germany; ^4^Department of Pediatrics, University Hospital Muenster, Muenster, Germany

**Keywords:** thrombectomy, pediatric, stroke, newborn, basilar, neonatal stroke

## Abstract

**Background:**

Neonatal stroke remains a rare condition that has not yet been assessed in the field of endovascular treatment.

**Case:**

We present the first case report of a successful mechanical thrombectomy in a newborn with a basilar occlusion the treatment was 14 hours after birth. Complete reperfusion of the basilar artery was achieved after the two thrombectomy maneuvers with stent retrievers. Imaging follow-up proved patency of the target vessel and at day 30, the patient showed no neurologic deficits.

**Conclusions:**

Mechanical thrombectomy appears to be technically feasible and can be an individual option in selected cases to treat stroke in neonates with proven persistent proximal cerebral artery occlusion.

## Introduction

Pediatric stroke, due to arterial occlusions, occurs rarely with the estimated incidences between 2 and 8 per 1,00,000 per year (1). Similar to adults, pediatric stroke carries a high risk of mortality and long term disability. While the evidence of mechanical thrombectomy (MT) for adults, with large vessel occlusion (LVO) stroke, is based on multiple randomized clinical trials, there is a general paucity of data for endovascular stroke treatment (EVT) of patients with pediatric stroke. Nonetheless, retrospective case series (2–4) suggest the feasibility and safety of the said procedure in childhood stroke from 7 years of age (2, 4). Currently, a successful MT has been reported in children as young as 9 months (5). There is no data on EVT in neonatal arterial ischemic stroke (NAIS) (5, 6).

Neonatal arterial ischemic stroke differs from childhood stroke in terms of underlying causes (7–9). The NAIS can be caused by placenta thrombembolism that is related to different risk factors (10). Most NAIS are presented with anterior circulation mainly in the middle cerebral artery (MCA) territory, whereas posterior circulation strokes are extremely rare. Furthermore, in most cases of NAIS, a vessel occlusion can not be proven at the time of diagnosis with imaging. To our knowledge, we present the first case of a successful MT for arterial stroke in a newborn, 14 h after birth.

## Case Description

### Clinical Presentation

The timeline with clinical and procedural data is shown in Table 1.

**Table 1:** Timeline of clinical and procedural data.

**0 h**	**Birth**
6 h	Clinical symptoms: movement abnormalities
10 h	Diagnostic MRI
13:30 h	Initiation of anesthesia, pre-interventional transcranial ultrasound
14 h	Interventional procedure
20 h	Transcranial ultrasound
Day 2	MRI
Day 16	MRI
3 months	Clinical and MRI follow up

A male mature neonate (41 weeks, 4,620 g body weight) was born per vacuum extraction, with an initial Appearance, Pulse, Grimace, Activity, Respiration (APGAR) Score of 7/8/9. Right after birth, respiratory adaption disturbance was observed, and the neonate was admitted to the pediatric intensive care unit. Six hours after birth, he presented movement abnormalities. As a differential diagnosis, seizures were suspected. Ultrasound showed no clear pathology, and an MRI imaging was immediately initiated for a suspected NAIS as a differential diagnosis.

### Diagnostic Assessment

**Figure 1:**
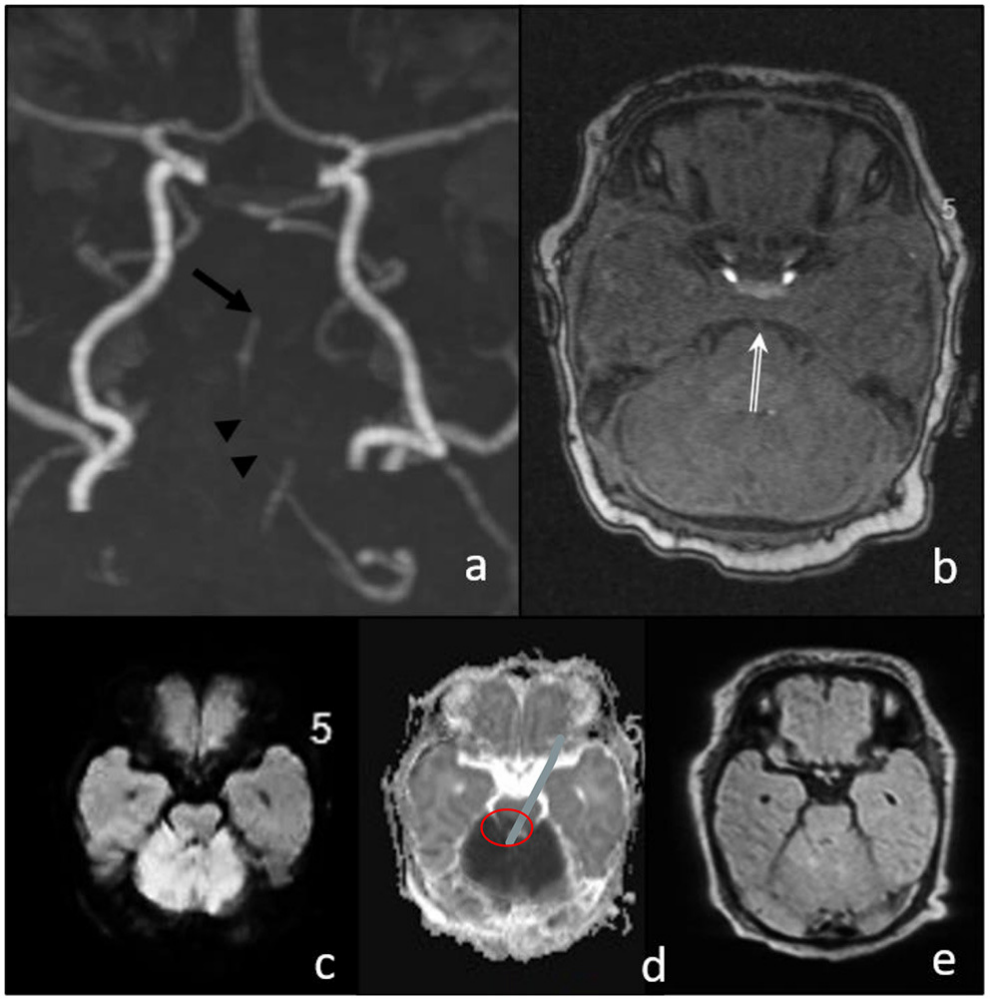
Magnetic resonance imaging (MRI) before intervention. **(a,b)** TOF shows the stop of flow signal in the distal basilar artery (arrow in a and double arrow in **(b)**, and a discontinuous flow signal in the proximal basilar artery (arrowheads); **(c)** diffusion-weighted imaging (DWI) MRI signal elevation and ADC decrease **(d)** in the cerebellum corresponding to SCA territory without significant FLAIR changes **(e)**. Only small diffusion restriction in the brain stem (medulla oblongata).

Magnetic resonance imaging (MRI) including MR angiography was performed 4 h after the onset of the symptom, demonstrating a distal basilar artery (BA) occlusion with bilateral lesions on diffusion-weighted imaging (DWI) in the territory of the superior cerebellar artery (Figure 1), and a very small and circumscribed brain stem lesion in the medulla oblongata.

The patient was immediately referred to our hospital for a potential EVT. After the arrival of the patient, intubation anesthesia was initiated. In the angio-suite, prior to the procedure, a transcranial ultrasound confirmed the persistence of the distal BA occlusion.

### Interventional Procedure

**Figure 2:**
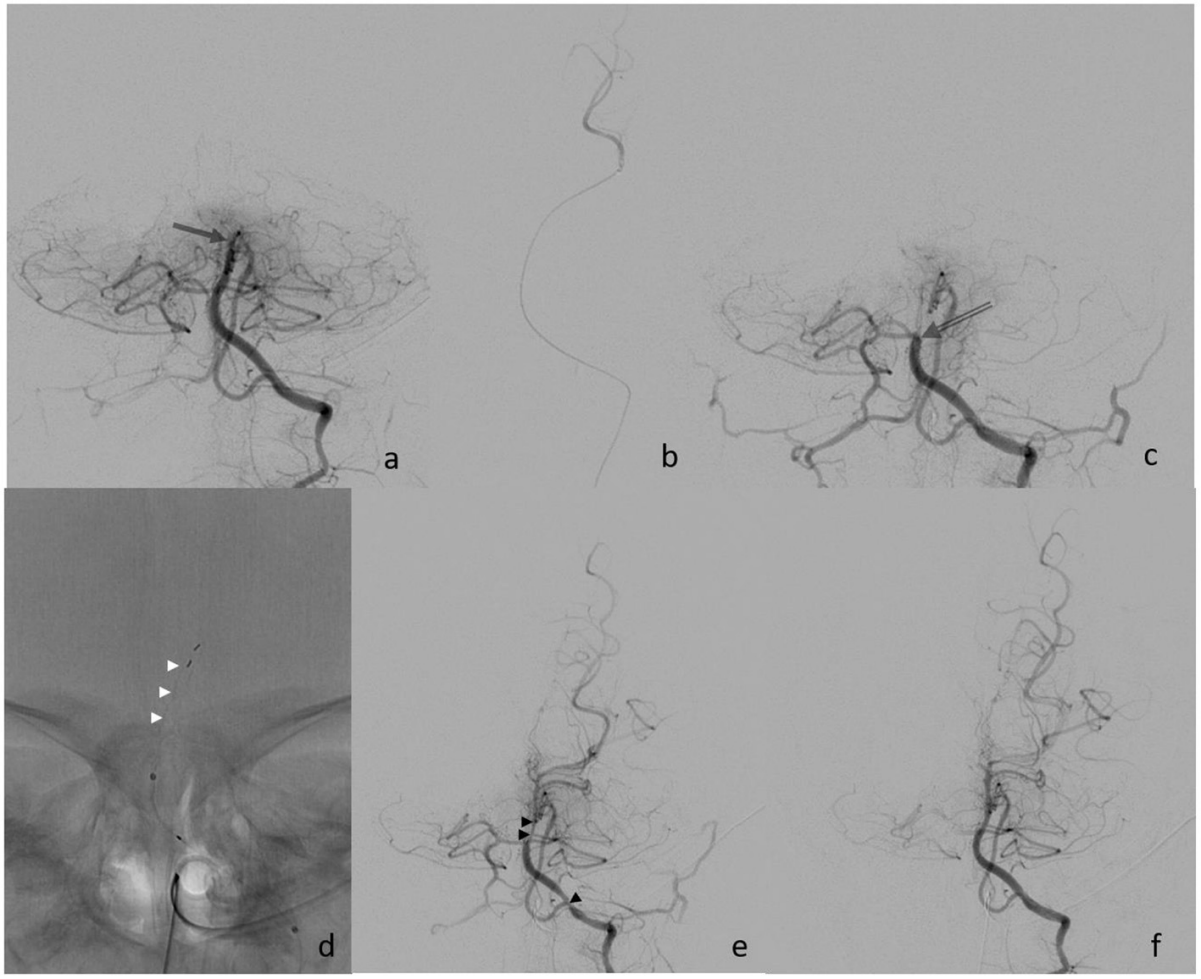
**(a)** Initial finding of distal basilar artery occlusion (arrow); **(b)** super selective injection into left PCA; **(c)** proximal occlusion after the 1st retriever maneuver (open arrow); **(d)** deployment of Preset device (white arrowheads); **(e)** finding after the 2nd retrieval with vasospasm (black arrowheads); and **(f)** final control after nimodipine infusion.

Eight hours after the symptom onset, the procedure started with the establishment of right femoral access using a 4-French (F) sheath. A 4F 100 cm vertebral shape catheter (Cordis Tempo, Cardinal Health, Dublin, Ireland) was used as a guiding catheter. The left vertebral artery (VA) showed a direct origin from the aortic arch and was then proximally catheterized. Bilateral subtraction angiography confirmed the distal occlusion of the hypoplastic BA (Figure 2). The BA was catheterized with a 156 cm Headway DUO microcatheter (Microvention, Aliso Vejo, CA, USA), guided by a manually pre-shaped Hybrid.008″ guidewire (Balt, Montmorency, France), and the thrombus passage was confirmed by a contrast injection showing a distal position in the left posterior cerebral artery (Figure 2b). MT was performed with a Catch Mini 3 ×15 mm (Balt, Montmorency, France) that was deployed within the occlusion. After 1 min, the stent retriever was pulled back toward the 4f-catheter and both devices were extracted under continuous aspiration. Subsequent catheterization of the left VA showed a persistent BA occlusion that was now more proximally located, and, therefore, a second MT maneuver was performed using a preset lite 3 ×20 mm (Phenox, Bochum Germany) device. Repeated catheterization of the left VA showed a completely recanalized but vasospastic BA. Within the next 5 min, a nimodipine infusion (0.1 mg) was applied through the catheter, followed by a final angiographic run that confirmed the visible improvement of the vasospasms and a patent BA 48 min after the start of the procedure, and 8 h after symptom onset.

### Clinical Development

**Figure 3:**
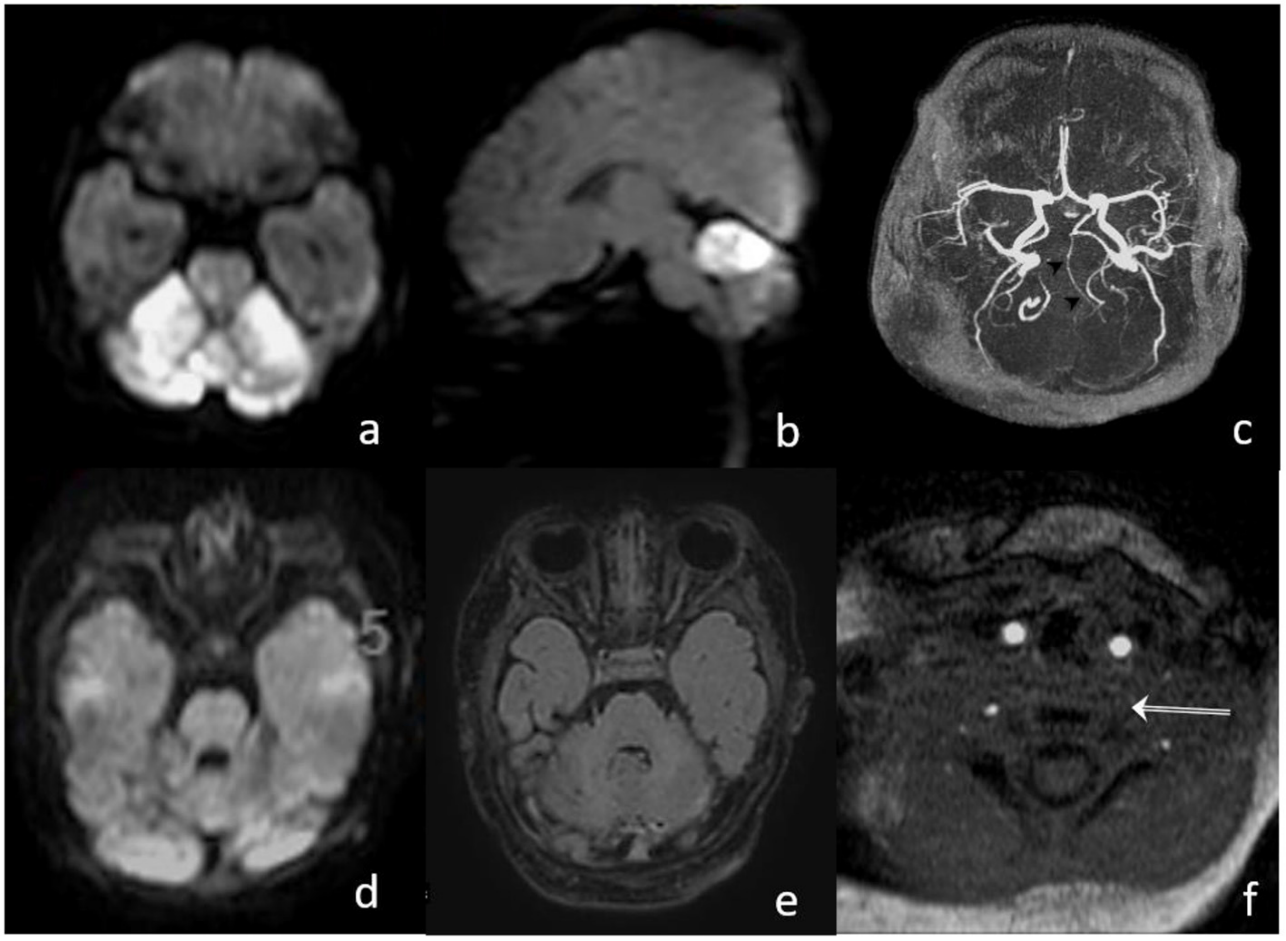
**(a,b)** MRI on day 1, after the intervention, shows bright DWI lesions in both SCA territories with small brain stem involvement; **(c)** TOF proves patent hypoplastic basilar artery; **(d,e)** MRI on day 15 shows normalization of DWI signal and decrease in the FLAIR lesion in the cerebellum; and **(f)** missing proximal flow signal in left VA.

Six hours postintervention, doppler ultrasound confirmed the recanalization of the BA with antegrade perfusion, and 24 h follow-up MRI angiography showed patency of the BA with a regular flow profile. There was no increase in the volume of DWI abnormalities (Figure 3). After the extubation, the clinical condition was stable with intermittent mild declines in oxygen saturation for 2 days. Medical therapy was continued with phenobarbital for 14 days and with nimodipine for 7 days, respectively, to prevent seizures and vasospasm. Additionally, enoxaparine was started once daily as an individual treatment, with a likely proximal left vertebral artery occlusion or low flow. The patient was discharged from our hospital on day 17, after the stroke with normal motoric patterns and with no neurological deficits.

Postinterventional follow-up imaging with MRI (day 1 and 15) and a transcranial ultrasound showed a proximal occlusion of the left VA, which had been catheterized during the intervention (Figure 3f). In an etiologic stroke workup, a small atrial septal defect was confirmed by a cardiac ultrasound. Clinical follow-up examination 30 days after the stroke showed a normal neurological examination.

**Figure 4:**
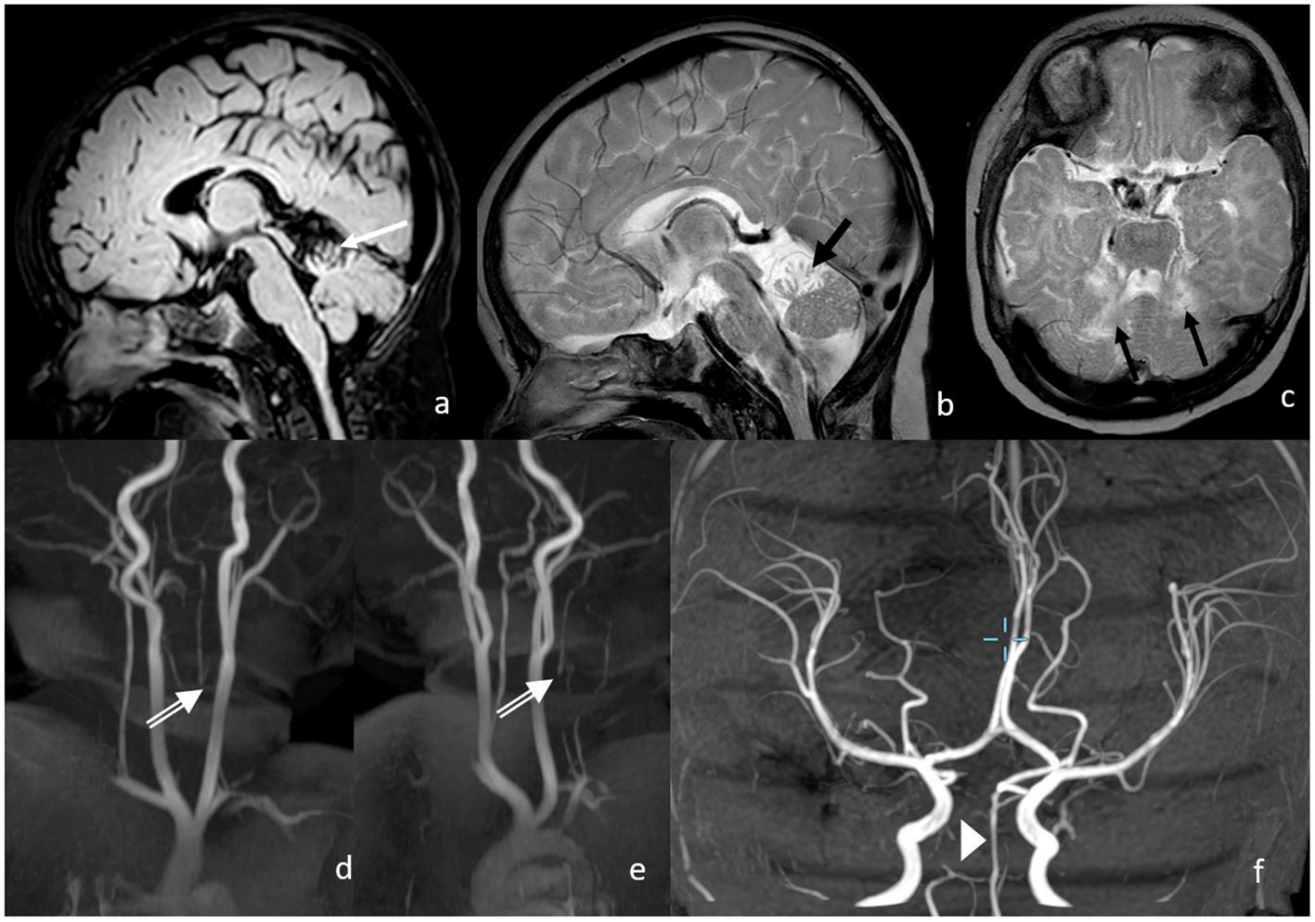
Three months following up of MRI and MRA. Postischemic defects in bilateral SCA territory with focal atrophy in the cerebellum on the following: **(a)** FLAIR (white arrow); **(b,c)** T2w (black arrows), proximal vertebral artery shows no antegrade flow in v0-V2 with distal reperfusion; **(d,e)** white double arrows; and **(f)** the basilar artery shows normal flow signal.

In the 3 months of the neurological follow-up examination, the evolution was appropriate to the age with a symmetrical activity of all the extremities. Intermittent strabismus, with abduction deficit of the right more than the left, was observed as well as a partially fixed head malposition with left rotation. The MRI showed a normal perfused basilar artery without signs of new ischemia (Figure 4). The cerebellum shows defects in the territory of the superior cerebellar artery with focal atrophy.

According to guidelines, detailed hemostaseologic testing was performed. The lipoprotein elevation was diagnosed as a potential risk factor for thromboembolic events in childhood and NAIS (11, 12).

## Discussion

Neonatal stroke due to arterial vessel occlusion is a rare condition, especially in cases with BA involvement (13). The management of perinatal stroke is usually conservative and supportive (14). Our reported case shows a very rare constellation of findings of perinatal ischemia with cerebellar infarction associated with a persistent thromboembolic occlusion of the basilar artery, in contrast to the more common NAIS in the anterior circulation, the latter is often without a proven arterial occlusion.

In our case, two contributing factors for NAIS were present: the diagnosis of an atrial septal defect and the elevated lipoprotein a. These factors may have provoked an embolic stroke. Additionally, the anatomical variant of the left VA, with a direct origin from the aortic arch, supported the hypothesis of an embolic BA occlusion. In our case, there were no typical signs for basilar artery occlusion known for older children such as lethargy or apnoe. This might be due to the different brain maturiation in a newborn. Unspecific symtoms and mimicking seizures lead to immediate imaging including MRI and surprisingly showed the mid-basilar occlusion.

The clinical constellation and the imaging findings indicated a high risk of progressive infarction and a potentially life-threatening condition. Accordingly, at the time of MRI diagnosis, the brainstem showed only one small DWI lesion suggesting a large part of the basilar territory to be a tissue at risk that could potentially be preserved with acute reperfusion of the occluded artery. The decision for MT after the MRI, as well as the secondary admission, was made in an interdisciplinary consensus with repeated proof of the persistent basilar occlusion by ultrasound in the Angiosuite prior to the procedure.

Attributed to the latest technical developments, the interventional procedure was facilitated by the availability of small clot retrievers dedicated for EVT in adults with medium-sized vessel occlusions like the distal posterior cerebral artery (15). However, there are several clinical and, maybe, even ethical concerns for and against EVT in this special context that have to be weighed-out based on a case-by-case basis with individual risk-benefit-ratios. Although the diagnosis and the secondary admission to our center were unusually fast (16), the procedure started with a considerable delay of 8 h after the symptom onset. Thus, the collateral blood flow of the vertebrobasilar territory might have been so well-preserved, that an EVT carried a risk to be unnecessary (collaterals will preserve the target tissue either way) or futile (tissue at risk is already irreversibly damaged), and, therefore, the procedure could favor possible harmful treatment effects (radiation, anesthesiologic complications, iatrogenic vessel injuries, and bleeding events). In our case, the postinterventional absence of flow in the left proximal VA may be explained due to a procedure-related vessel dissection caused by repetitive catheterization of the small vessel with a relatively stiff 4F-catheter. Notwithstanding the latter, endovascular therapy for neonates is well-established in the fields of cardiology and neuroradiology (17–20). The evolution of stroke therapy in adults, with rapid advances in device technology, supports the MT of neonates with LVO as a potential frontier in interventional neuroradiology. Besides the technical equipment, however, an effective EVT of NAIS requires a high-paced workflow (“time is brain”) that can only be achieved in a multidisciplinary environment with highly specialized and trained teams in pediatrics, anesthesiology, and interventional neuroradiology. In such a setting, the in-hospital time metrics of our case were comparable to the large multicenter adult cohorts with a median door-to-groin puncture time of 55 min, marking an important predictor of a favorable outcome (21). Due to the rarity of neonatal LVO stroke, prospective trials will be very challenging to conduct, and, currently, decision-making in treatment depends on the individual expertise of the multidisciplinary team and of the individual case. Our reported experience is limited to the particular findings of this case and is not directly applicable to the common NAIS scenario in the anterior circulation.

## Conclusion

In our first case of postnatal basilar artery occlusion stroke, mechanical thrombectomy was technically feasible and successful with a good clinical outcome. Endovascular therapy might be an option for selected NAIS with certain findings.

## Data Availability Statement

The original contributions presented in the study are included in the article/supplementary material, further inquiries can be directed to the corresponding author/s.

## Ethics Statement

The studies involving human participants were reviewed and approved by Aerztekammer Westfalen Lippe. Written informed consent to participate in this study was provided by the participants' legal guardian/next of kin. Written informed consent was obtained from the individual(s) and minor(s)' legal guardian/next of kin, for the publication of any potentially identifiable images or data included in this article.

## Author Contributions

CS indicated and performed the interventional procedure. LM wrote/edited the manuscript and data. WS performed the interventional procedure and image processing. AR initiated the case and performed imaging. RS performed clinically and follow-up exams and edited the manuscript. All authors contributed to the article and approved the submitted version.

## Conflict of Interest

The authors declare that the research was conducted in the absence of any commercial or financial relationships that could be construed as a potential conflict of interest.

## Publisher's Note

All claims expressed in this article are solely those of the authors and do not necessarily represent those of their affiliated organizations, or those of the publisher, the editors and the reviewers. Any product that may be evaluated in this article, or claim that may be made by its manufacturer, is not guaranteed or endorsed by the publisher.

## Abbreviations

BA: Basilar Artery
DWI: Diffusion-Weighted Imaging
EVT: Endovascular Treatment
F: French
LVO: Lage Vessel Occlusion
MT: Mechanical Thrombectomy
MRI: Magnetic Resonance Imaging
NAIS: Neonatal arterial ischemic stroke
VA: Vertebral Artery.

## References

[1] Mallick AA Ganesan V Kirkham FJ Fallon P Hedderly T McShane T. Childhood arterial ischaemic stroke incidence, presenting features, and risk factors: a prospective population-based study. Lancet Neurol. (2014) 13:35–43. 10.1016/S1474-4422(13)70290-4 24304598

[2] Bhatia K Kortman H Blair C Parker G Brunacci D Ang T. Mechanical thrombectomy in pediatric stroke: systematic review, individual patient data meta-analysis, and case series. J Neurosurg Pediatrics. (2019) 24:558–71. 10.3171/2019.5.PEDS19126 31398697

[3] Bigi S Dulcey A Gralla J Bernasconi C Melliger A Datta AN. Feasibility, safety, and outcome of recanalization treatment in childhood stroke. Ann Neurol. (2018) 83:1125–32. 10.1002/ana.25242 29679441

[4] Sporns PB Sträter R Minnerup J Wiendl H Hanning U Chapot R. Feasibility, safety, and outcome of endovascular recanalization in childhood stroke. Jama Neurol. (2020) 77:25–34. 10.1001/jamaneurol.2019.3403 31609380 PMC6802048

[5] Sun LR Felling RJ Pearl MS. Endovascular mechanical thrombectomy for acute stroke in young children. J Neurointervent Surg. (2019) 11:554. 10.1136/neurintsurg-2018-014540 30842305

[6] Nicosia G Cicala D Mirone G. Childhood acute basilar artery thrombosis successfully treated with mechanical thrombectomy using stent retrievers: case report and review of the literature. Child's Nerv Syst. (2017) 33:349–355. 10.1007/s00381-016-3259-z 27704247

[7] De Veber GA Kirton A Booth FA Yager JY Wirrell EC Wood E. Epidemiology and outcomes of arterial ischemic stroke in children: the Canadian pediatric ischemic stroke registry. Pediatr Neurol. (2017) 69:58–70. 10.1016/j.pediatrneurol.2017.01.016 28254555

[8] Golomb MR MacGregor DL Domi T. Presumed pre- or perinatal arterial ischemic stroke: risk factors and outcomes. Ann Neurol. (2001) 50:163–8. 10.1002/ana.1078 11506398

[9] Kirton A Armstrong-Wells J Chang T Deveber G Rivkin MJ Hernandez M. Symptomatic neonatal arterial ischemic stroke: the international pediatric stroke study. Pediatrics. (2011) 128:e1402–10. 10.1542/peds.2011-1148 22123886

[10] Fluss J Dinomais M Chabrier S. Perinatal stroke syndromes: Similarities and diversities in aetiology, outcome and management. Eur J Paediatr Neurol. (2019) 23:368–83. 10.1016/j.ejpn.2019.02.013 30879961

[11] Günther G Junker R Stra?ter R Schobess R Kurnik K Kosch A. Symptomatic ischemic stroke in full-term neonates: role of acquired and genetic prothrombotic risk factors. Stroke. (2000) 31:2437–41. 10.1161/01.STR.31.10.2437 11022077

[12] Kenet G Lütkhoff LK Albisetti M Bernard T Bonduel M Brandao L. Impact of thrombophilia on risk of arterial ischemic stroke or cerebral sinovenous thrombosis in neonates and children: a systematic review and meta-analysis of observational studies. Circulation. (2010) 121:1838–47. 10.1161/CIRCULATIONAHA.109.913673 20385928

[13] Govaert P Dudink J Visser G Breukhoven P Vanhatalo S Lequin M. Top of the basilar artery embolic stroke and neonatal myoclonus. Dev Med Child Neurol. (2009) 51:324–7. 10.1111/j.1469-8749.2008.03183.x 19207294

[14] Armstrong-Wells J Ferriero DM. Diagnosis and acute management of perinatal arterial ischemic stroke. Neurol Clin Pract. (2014) 4:378–85. 10.1212/CPJ.0000000000000077 25317375 PMC4196460

[15] Meyer L Stracke CP Jungi N Wallocha M Broocks G Sporns PB. Thrombectomy for primary distal posterior cerebral artery occlusion stroke: the TOPMOST study. JAMA Neurol. (2021) 21:1. 10.1001/jamaneurol.2021.0001 33616642 PMC7900924

[16] Rollins N Pride GL Plumb PA Dowling MM. Brainstem strokes in children: an 11-year series from a tertiary pediatric center. Pediatr Neurol. (2013) 49:458–64. 10.1016/j.pediatrneurol.2013.07.007 24080274

[17] Arya A Ranjan R Pushkarna R. Neonatal axillary artery thrombosis requiring emergent thrombectomy. Indian J Pediatrics. (2021) 21:1–2. 10.1007/s12098-020-03585-5 33415548

[18] Patel ND Takao C Badran S Sullivan PM. Neonatal arterial thrombosis: treatment *via* a patent ductus arteriosus. Prog Pediatr Cardiol. (2021) 2021:101374. 10.1016/j.ppedcard.2021.101374

[19] Zelenak K Uhrikova Z Mikler J Zibolen M. Mechanical thrombectomy for cerebral venous sinus thrombosis in a neonate. Indian Pediatr. (2020) 57:862–3. 10.1007/s13312-020-1971-y 32999120

[20] Brinjikji W Krings T Murad MH Rouchaud A Meila D. Endovascular treatment of vein of galen malformations: a systematic review and meta-analysis. AJNR Am J Neuroradiol. (2017) 38:2308–2314. 10.3174/ajnr.A5403 28982789 PMC7963723

[21] Jahan R Saver JL Schwamm LH Fonarow GC Liang L Matsouaka RA. Association between time to treatment with endovascular reperfusion therapy and outcomes in patients with acute ischemic stroke treated in clinical practice. JAMA. (2019) 322:252–63. 10.1001/jama.2019.8286 31310296 PMC6635908

